# Triethyl­ammonium tetra­chlorido(pyrazine-2-carboxyl­ato-κ^2^ *N* ^1^,*O*)stannate(IV)

**DOI:** 10.1107/S1600536811016473

**Published:** 2011-05-07

**Authors:** Ezzatollah Najafi, Mostafa M. Amini, Seik Weng Ng

**Affiliations:** a Department of Chemistry, General Campus, Shahid Beheshti University, Tehran 1983963113, Iran; b Department of Chemistry, University of Malaya, 50603 Kuala Lumpur, Malaysia

## Abstract

The Sn^IV^ atom in the title ammonium stannate, (Et_3_NH)[Sn(C_5_H_3_N_2_O_2_)Cl_4_], is chelated by an pyrazine-2-carboxyl­ate ligand and exists in a *cis*-SnCl_4_NO octa­hedral geometry. The cation and the anion are linked by an N—H⋯N hydrogen bond.

## Related literature

For triethyl­ammonium tetra­chlorido(pyridine-2-carboxyl­ato)stannate(IV), see: Najafi *et al.* (2011 ▶).
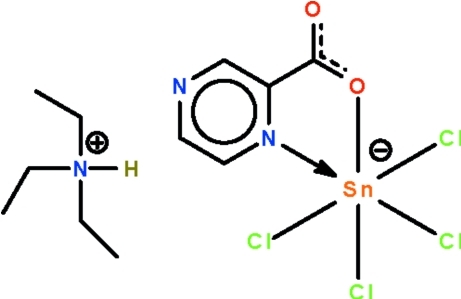


## Experimental

### 

#### Crystal data

(C_6_H_16_N)[Sn(C_5_H_3_N_2_O_2_)Cl_4_]
*M* *_r_* = 485.78
Triclinic, 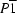

*a* = 7.4497 (2) Å
*b* = 9.9752 (3) Å
*c* = 12.3728 (4) Å
α = 86.491 (2)°
β = 80.125 (3)°
γ = 83.817 (2)°
*V* = 899.70 (5) Å^3^
*Z* = 2
Mo *K*α radiation
μ = 2.02 mm^−1^
*T* = 100 K
0.35 × 0.30 × 0.25 mm

#### Data collection

Agilent SuperNova Dual diffractometer with an Atlas detector
Absorption correction: multi-scan (*CrysAlis PRO*; Agilent, 2010 ▶) *T*_min_ = 0.538, *T*_max_ = 0.632
15321 measured reflections
4094 independent reflections
3800 reflections with *I* > 2σ(*I*)
*R*_int_ = 0.030

#### Refinement

*R*[*F* ^2^ > 2σ(*F* ^2^)] = 0.022
*wR*(*F* ^2^) = 0.052
*S* = 1.02
4094 reflections
194 parameters
H atoms treated by a mixture of independent and constrained refinement
Δρ_max_ = 0.35 e Å^−3^
Δρ_min_ = −0.65 e Å^−3^

### 

Data collection: *CrysAlis PRO* (Agilent, 2010 ▶); cell refinement: *CrysAlis PRO*; data reduction: *CrysAlis PRO*; program(s) used to solve structure: *SHELXS97* (Sheldrick, 2008 ▶); program(s) used to refine structure: *SHELXL97* (Sheldrick, 2008 ▶); molecular graphics: *X-SEED* (Barbour, 2001 ▶); software used to prepare material for publication: *publCIF* (Westrip, 2010 ▶).

## Supplementary Material

Crystal structure: contains datablocks global, I. DOI: 10.1107/S1600536811016473/qk2007sup1.cif

Structure factors: contains datablocks I. DOI: 10.1107/S1600536811016473/qk2007Isup2.hkl

Additional supplementary materials:  crystallographic information; 3D view; checkCIF report

## Figures and Tables

**Table 1:** Hydrogen-bond geometry (Å, °)

*D*—H⋯*A*	*D*—H	H⋯*A*	*D*⋯*A*	*D*—H⋯*A*
N3—H1⋯N2	0.91 (3)	2.10 (3)	2.999 (2)	167 (2)

**Fig. 1.**
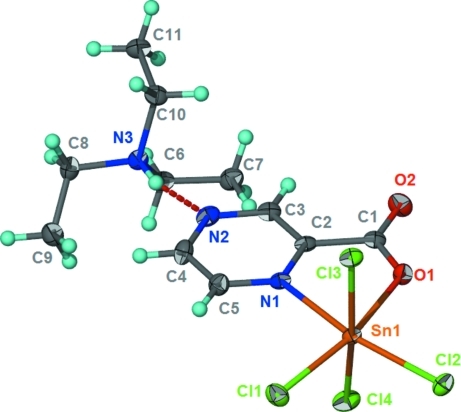
Thermal ellipsoid plot (Barbour, 2001) of (Et3NH)+ [SnCl4(C5H3N2O2)]- at the 70% probability level; hydrogen atoms are drawn as spheres of arbitrary radius. The hydrogen bond is denoted by a dashed bond.

## supplementary crystallographic information

### Comment

We have recently reported the crystal structure of triethylammonium tetrachlorido(pyridine-2-carboxylato)stannate, which was synthesized by the reaction of triethylammonium pyridine-2-carboxylate and stannic chloride. The Sn^IV^ atom in the anion is *N*,*O*-chelated by the pyridine-2-carboxylate in a *cis*-SnCl_4_NO octahedral geometry (Najafi *et al.*, 2011). In our previous studies, we have reacted aromatic carboxylic acid with stannic chloride, with/without a proton-abstraction agent. In the present study, replacing pyrdine-2-carboxylic acid by pyrazine-2-carboxylic acid affords a similar salt, (Et_3_NH)^+^ [SnCl_4_(C_5_H_3_N_2_O_2_)]^-^ (Scheme I, Fig. 1). The tin atom in the stannate is chelated by the pyrazine-2-carboxylate group in a *cis*-SnCl_4_NO octahedral geometry. The cation forms an N–H···N hydrogen bond with the anion. Of the four Sn–Cl bonds, the ones that are *trans* to the Sn–O/Sn–N bonds are somewhat shorter than the other two. No Cl···Cl interactions are present.

### Experimental

The reaction was carried out under a nitrogen atmosphere. Pyrazine-2-carboxylic acid (1.0 mmol, 0.12 g) and the triethylamine (1.0 mmol, 0.10 g) were dissolved in dry methanol (20 ml). Stannic chloride (1.0 mmol, 0.35 g) was added to the mixture and stirred for 12 h. Suitable crystals were obtained by slow evaporation of the solvent.

### Refinement

Carbon-bound H-atoms were placed in calculated positions (C—H 0.95 to 0.98 Å) and were included in the refinement in the riding model approximation, with *U*(H) set to 1.2 to 1.5*U*(C). The ammonium H-atom was located in a difference Fourier map, and was freely refined.

### Crystal data

**Table:**

(C_6_H_16_N)[Sn(C_5_H_3_N_2_O_2_)Cl_4_]	*Z* = 2
*M**_r_* = 485.78	*F*(000) = 480
Triclinic, *P*1	*D*_x_ = 1.793 Mg m^−^^3^
Hall symbol: -P 1	Mo *K*α radiation, λ = 0.71073 Å
*a* = 7.4497 (2) Å	Cell parameters from 10335 reflections
*b* = 9.9752 (3) Å	θ = 2.6–29.2°
*c* = 12.3728 (4) Å	µ = 2.02 mm^−^^1^
α = 86.491 (2)°	*T* = 100 K
β = 80.125 (3)°	Irregular block, colorless
γ = 83.817 (2)°	0.35 × 0.30 × 0.25 mm
*V* = 899.70 (5) Å^3^	

### Data collection

**Table:**

Agilent SuperNova Dual diffractometer with an Atlas detector	4094 independent reflections
Radiation source: SuperNova (Mo) X-ray Source	3800 reflections with *I* > 2σ(*I*)
Mirror	*R*_int_ = 0.030
Detector resolution: 10.4041 pixels mm^-1^	θ_max_ = 27.5°, θ_min_ = 2.6°
ω scans	*h* = −9→9
Absorption correction: multi-scan (*CrysAlis PRO*; Agilent, 2010)	*k* = −12→12
*T*_min_ = 0.538, *T*_max_ = 0.632	*l* = −15→16
15321 measured reflections	

### Refinement

**Table:**

Refinement on *F*^2^	Primary atom site location: structure-invariant direct methods
Least-squares matrix: full	Secondary atom site location: difference Fourier map
*R*[*F*^2^ > 2σ(*F*^2^)] = 0.022	Hydrogen site location: inferred from neighbouring sites
*wR*(*F*^2^) = 0.052	H atoms treated by a mixture of independent and constrained refinement
*S* = 1.02	*w* = 1/[σ^2^(*F*_o_^2^) + (0.0257*P*)^2^ + 0.2896*P*] where *P* = (*F*_o_^2^ + 2*F*_c_^2^)/3
4094 reflections	(Δ/σ)_max_ = 0.001
194 parameters	Δρ_max_ = 0.35 e Å^−^^3^
0 restraints	Δρ_min_ = −0.65 e Å^−^^3^

### Fractional atomic coordinates and isotropic or equivalent isotropic displacement parameters (Å^2^)

**Table:**

	*x*	*y*	*z*	*U*_iso_*/*U*_eq_	
Sn1	0.310753 (18)	0.248889 (13)	0.740250 (10)	0.01198 (5)	
Cl1	0.46068 (7)	0.34171 (5)	0.86858 (4)	0.01988 (11)	
Cl2	0.00680 (7)	0.26586 (5)	0.83792 (4)	0.01703 (11)	
Cl3	0.39067 (7)	0.02423 (5)	0.80641 (4)	0.01605 (11)	
Cl4	0.26028 (7)	0.46373 (5)	0.64356 (4)	0.02064 (11)	
O1	0.22917 (19)	0.16094 (14)	0.60971 (11)	0.0149 (3)	
O2	0.3047 (2)	0.10363 (15)	0.43513 (11)	0.0194 (3)	
N1	0.5661 (2)	0.22659 (16)	0.61249 (13)	0.0128 (3)	
N2	0.8334 (2)	0.21721 (17)	0.42643 (14)	0.0171 (4)	
N3	1.0881 (2)	0.24246 (17)	0.21208 (14)	0.0142 (3)	
C1	0.3419 (3)	0.14663 (19)	0.51771 (16)	0.0134 (4)	
C2	0.5317 (3)	0.18609 (19)	0.51747 (16)	0.0128 (4)	
C3	0.6667 (3)	0.18070 (19)	0.42525 (16)	0.0155 (4)	
H3A	0.6397	0.1501	0.3592	0.019*	
C4	0.8662 (3)	0.2544 (2)	0.52239 (17)	0.0179 (4)	
H4A	0.9846	0.2789	0.5264	0.021*	
C5	0.7345 (3)	0.2587 (2)	0.61660 (17)	0.0168 (4)	
H5A	0.7638	0.2844	0.6837	0.020*	
C6	0.9980 (3)	0.3321 (2)	0.13009 (16)	0.0161 (4)	
H6A	1.0734	0.3221	0.0564	0.019*	
H6B	0.9943	0.4272	0.1495	0.019*	
C7	0.8062 (3)	0.3011 (2)	0.12490 (18)	0.0201 (5)	
H7A	0.7565	0.3610	0.0691	0.030*	
H7B	0.7288	0.3151	0.1966	0.030*	
H7C	0.8084	0.2070	0.1057	0.030*	
C8	1.2722 (3)	0.2874 (2)	0.22036 (17)	0.0173 (4)	
H8A	1.3358	0.3118	0.1459	0.021*	
H8B	1.3477	0.2116	0.2511	0.021*	
C9	1.2544 (3)	0.4075 (2)	0.29232 (19)	0.0220 (5)	
H9A	1.3763	0.4344	0.2947	0.033*	
H9B	1.1952	0.3827	0.3668	0.033*	
H9C	1.1801	0.4829	0.2620	0.033*	
C10	1.1019 (3)	0.0937 (2)	0.19176 (16)	0.0168 (4)	
H10A	1.1673	0.0429	0.2470	0.020*	
H10B	0.9769	0.0646	0.2021	0.020*	
C11	1.2003 (3)	0.0582 (2)	0.07822 (17)	0.0189 (4)	
H11A	1.2039	−0.0392	0.0701	0.028*	
H11B	1.3255	0.0840	0.0681	0.028*	
H11C	1.1352	0.1067	0.0230	0.028*	
H1	1.015 (3)	0.249 (2)	0.279 (2)	0.027 (7)*	

### Atomic displacement parameters (Å^2^)

**Table:**

	*U*^11^	*U*^22^	*U*^33^	*U*^12^	*U*^13^	*U*^23^
Sn1	0.01291 (8)	0.01297 (8)	0.00947 (8)	0.00012 (5)	−0.00092 (5)	−0.00091 (5)
Cl1	0.0232 (3)	0.0225 (3)	0.0154 (2)	−0.0057 (2)	−0.0037 (2)	−0.0045 (2)
Cl2	0.0141 (2)	0.0218 (3)	0.0137 (2)	0.00087 (19)	0.00009 (19)	−0.00038 (19)
Cl3	0.0189 (3)	0.0141 (2)	0.0142 (2)	0.00126 (19)	−0.00218 (19)	0.00034 (18)
Cl4	0.0249 (3)	0.0158 (2)	0.0181 (2)	0.0031 (2)	0.0002 (2)	0.00323 (19)
O1	0.0143 (7)	0.0201 (7)	0.0107 (7)	−0.0020 (6)	−0.0023 (6)	−0.0023 (6)
O2	0.0241 (8)	0.0229 (8)	0.0123 (7)	−0.0054 (6)	−0.0030 (6)	−0.0027 (6)
N1	0.0154 (9)	0.0120 (8)	0.0104 (8)	0.0013 (7)	−0.0017 (7)	−0.0008 (6)
N2	0.0182 (9)	0.0166 (9)	0.0150 (9)	0.0021 (7)	−0.0011 (7)	0.0008 (7)
N3	0.0151 (9)	0.0164 (9)	0.0103 (8)	−0.0020 (7)	0.0002 (7)	−0.0004 (7)
C1	0.0183 (10)	0.0092 (9)	0.0121 (9)	0.0000 (8)	−0.0028 (8)	0.0015 (7)
C2	0.0167 (10)	0.0088 (9)	0.0116 (9)	0.0016 (7)	−0.0011 (8)	0.0006 (7)
C3	0.0201 (11)	0.0130 (10)	0.0126 (10)	0.0001 (8)	−0.0023 (8)	0.0004 (8)
C4	0.0133 (10)	0.0222 (11)	0.0180 (10)	−0.0011 (8)	−0.0038 (8)	0.0028 (8)
C5	0.0157 (10)	0.0189 (10)	0.0161 (10)	−0.0010 (8)	−0.0040 (8)	−0.0005 (8)
C6	0.0195 (11)	0.0144 (10)	0.0139 (10)	0.0010 (8)	−0.0035 (8)	0.0002 (8)
C7	0.0199 (11)	0.0211 (11)	0.0190 (10)	0.0015 (9)	−0.0046 (9)	−0.0012 (9)
C8	0.0140 (10)	0.0213 (11)	0.0164 (10)	−0.0028 (8)	−0.0017 (8)	0.0005 (8)
C9	0.0199 (11)	0.0191 (11)	0.0283 (12)	−0.0030 (9)	−0.0062 (10)	−0.0019 (9)
C10	0.0207 (11)	0.0135 (10)	0.0158 (10)	−0.0022 (8)	−0.0022 (9)	0.0009 (8)
C11	0.0215 (11)	0.0176 (11)	0.0169 (10)	0.0008 (9)	−0.0018 (9)	−0.0031 (8)

### Geometric parameters (Å, °)

**Table:**

Sn1—O1	2.0911 (13)	C4—H4A	0.9500
Sn1—N1	2.2558 (16)	C5—H5A	0.9500
Sn1—Cl2	2.3707 (5)	C6—C7	1.507 (3)
Sn1—Cl1	2.3742 (5)	C6—H6A	0.9900
Sn1—Cl3	2.3879 (5)	C6—H6B	0.9900
Sn1—Cl4	2.4150 (5)	C7—H7A	0.9800
O1—C1	1.299 (2)	C7—H7B	0.9800
O2—C1	1.217 (2)	C7—H7C	0.9800
N1—C5	1.337 (3)	C8—C9	1.517 (3)
N1—C2	1.340 (2)	C8—H8A	0.9900
N2—C3	1.334 (3)	C8—H8B	0.9900
N2—C4	1.334 (3)	C9—H9A	0.9800
N3—C6	1.508 (3)	C9—H9B	0.9800
N3—C8	1.509 (3)	C9—H9C	0.9800
N3—C10	1.510 (3)	C10—C11	1.513 (3)
N3—H1	0.91 (3)	C10—H10A	0.9900
C1—C2	1.508 (3)	C10—H10B	0.9900
C2—C3	1.385 (3)	C11—H11A	0.9800
C3—H3A	0.9500	C11—H11B	0.9800
C4—C5	1.389 (3)	C11—H11C	0.9800
			
O1—Sn1—N1	75.62 (6)	N1—C5—H5A	120.1
O1—Sn1—Cl2	91.17 (4)	C4—C5—H5A	120.1
N1—Sn1—Cl2	166.13 (4)	C7—C6—N3	113.48 (17)
O1—Sn1—Cl1	168.99 (4)	C7—C6—H6A	108.9
N1—Sn1—Cl1	93.47 (4)	N3—C6—H6A	108.9
Cl2—Sn1—Cl1	99.813 (18)	C7—C6—H6B	108.9
O1—Sn1—Cl3	86.56 (4)	N3—C6—H6B	108.9
N1—Sn1—Cl3	88.14 (4)	H6A—C6—H6B	107.7
Cl2—Sn1—Cl3	95.397 (18)	C6—C7—H7A	109.5
Cl1—Sn1—Cl3	91.748 (18)	C6—C7—H7B	109.5
O1—Sn1—Cl4	87.08 (4)	H7A—C7—H7B	109.5
N1—Sn1—Cl4	82.96 (4)	C6—C7—H7C	109.5
Cl2—Sn1—Cl4	92.285 (18)	H7A—C7—H7C	109.5
Cl1—Sn1—Cl4	93.058 (19)	H7B—C7—H7C	109.5
Cl3—Sn1—Cl4	170.120 (17)	N3—C8—C9	111.95 (17)
C1—O1—Sn1	119.66 (12)	N3—C8—H8A	109.2
C5—N1—C2	118.45 (17)	C9—C8—H8A	109.2
C5—N1—Sn1	129.40 (13)	N3—C8—H8B	109.2
C2—N1—Sn1	111.85 (13)	C9—C8—H8B	109.2
C3—N2—C4	116.56 (18)	H8A—C8—H8B	107.9
C6—N3—C8	110.51 (15)	C8—C9—H9A	109.5
C6—N3—C10	114.20 (15)	C8—C9—H9B	109.5
C8—N3—C10	111.59 (16)	H9A—C9—H9B	109.5
C6—N3—H1	108.5 (15)	C8—C9—H9C	109.5
C8—N3—H1	108.8 (15)	H9A—C9—H9C	109.5
C10—N3—H1	102.8 (15)	H9B—C9—H9C	109.5
O2—C1—O1	124.81 (19)	N3—C10—C11	113.47 (16)
O2—C1—C2	119.63 (17)	N3—C10—H10A	108.9
O1—C1—C2	115.56 (16)	C11—C10—H10A	108.9
N1—C2—C3	120.55 (18)	N3—C10—H10B	108.9
N1—C2—C1	116.58 (17)	C11—C10—H10B	108.9
C3—C2—C1	122.87 (17)	H10A—C10—H10B	107.7
N2—C3—C2	121.97 (18)	C10—C11—H11A	109.5
N2—C3—H3A	119.0	C10—C11—H11B	109.5
C2—C3—H3A	119.0	H11A—C11—H11B	109.5
N2—C4—C5	122.67 (19)	C10—C11—H11C	109.5
N2—C4—H4A	118.7	H11A—C11—H11C	109.5
C5—C4—H4A	118.7	H11B—C11—H11C	109.5
N1—C5—C4	119.72 (18)		
			
N1—Sn1—O1—C1	−6.70 (13)	C5—N1—C2—C1	178.09 (16)
Cl2—Sn1—O1—C1	169.01 (13)	Sn1—N1—C2—C1	−7.6 (2)
Cl1—Sn1—O1—C1	−14.2 (3)	O2—C1—C2—N1	−176.95 (18)
Cl3—Sn1—O1—C1	−95.65 (13)	O1—C1—C2—N1	2.4 (3)
Cl4—Sn1—O1—C1	76.78 (13)	O2—C1—C2—C3	2.6 (3)
O1—Sn1—N1—C5	−178.97 (18)	O1—C1—C2—C3	−178.07 (18)
Cl2—Sn1—N1—C5	162.83 (14)	C4—N2—C3—C2	2.5 (3)
Cl1—Sn1—N1—C5	−0.41 (17)	N1—C2—C3—N2	−1.1 (3)
Cl3—Sn1—N1—C5	−92.05 (17)	C1—C2—C3—N2	179.39 (18)
Cl4—Sn1—N1—C5	92.25 (17)	C3—N2—C4—C5	−1.5 (3)
O1—Sn1—N1—C2	7.47 (12)	C2—N1—C5—C4	2.4 (3)
Cl2—Sn1—N1—C2	−10.7 (3)	Sn1—N1—C5—C4	−170.79 (14)
Cl1—Sn1—N1—C2	−173.97 (12)	N2—C4—C5—N1	−1.0 (3)
Cl3—Sn1—N1—C2	94.39 (13)	C8—N3—C6—C7	−174.78 (16)
Cl4—Sn1—N1—C2	−81.31 (12)	C10—N3—C6—C7	58.4 (2)
Sn1—O1—C1—O2	−175.82 (15)	C6—N3—C8—C9	79.8 (2)
Sn1—O1—C1—C2	4.8 (2)	C10—N3—C8—C9	−151.96 (17)
C5—N1—C2—C3	−1.4 (3)	C6—N3—C10—C11	56.1 (2)
Sn1—N1—C2—C3	172.92 (15)	C8—N3—C10—C11	−70.1 (2)

### Hydrogen-bond geometry (Å, °)

**Table:**

*D*—H···*A*	*D*—H	H···*A*	*D*···*A*	*D*—H···*A*
N3—H1···N2	0.91 (3)	2.10 (3)	2.999 (2)	167 (2)
